# Self-care time and rating of health state in persons with diabetes: results from the population-based KORA survey in Germany

**DOI:** 10.1186/s12955-022-02068-9

**Published:** 2022-12-14

**Authors:** Andrea Icks, Simon Stöbel, Barbara Thorand, Rolf Holle, Michael Laxy, Michaela Schunk, Anja Neumann, Jürgen Wasem, Veronika Gontscharuk, Nadja Chernyak

**Affiliations:** 1 Institute for Health Services Research and Health Economics, Centre for Health and Society, Medical Faculty and University Hospital Düsseldorf, Heinrich-Heine-University Düsseldorf, Düsseldorf, Germany, Moorenstr. 5, 40225 Düsseldorf, Germany; 2 Institute for Health Services Research and Health Economics, German Diabetes Center, Leibniz Center for Diabetes Research at Heinrich-Heine-University Düsseldorf, Düsseldorf, Germany, Düsseldorf, Germany; 3 German Center for Diabetes Research, Partner Düsseldorf, München-Neuherberg, Germany, Munich-Neuherberg, Germany; 4 Institute of Epidemiology, Helmholtz Zentrum München, German Research Center for Environmental Health (GmbH), Neuherberg, Germany; 5 Faculty of Medicine, Institute for Medical Information Processing, Biometry and Epidemiology, LMU Munich, Munich, Germany; 6 Department for Public Health and Prevention, Faculty of Sports and Health Sciences, TUM Munich, Munich, Germany; 7 Katholische Stiftungshochschule München (KSH München) University of Applied Sciences, Munich, Germany; 8 Institute for Health Care Management and Research, University of Duisburg-Essen, Essen, Germany

**Keywords:** Patient time use, Diabetes mellitus, Health economic evaluation, population-based study

## Abstract

**Background:**

The amount of empirical research on whether people in fact include health-related changes in leisure time into health state valuations is limited and the results are inconclusive. In this exploratory study, we analyse whether time aspects of diabetes self-care might explain the ratings of the health state (HSR) in addition to the effects of physical and mental health-related quality of life.

**Methods:**

Using the data from participants with diagnosed type 2 diabetes in the population-based KORA FF4 study (*n* = 190, 60% Male, mean age 69 ± 10 years), multiple logistic regression models were fitted to explain HSR (good vs. poor) in terms of the SF-12 physical and mental component summary (PCS and MCS) scores, time spent on diabetes self-care and a number of background variables.

**Results:**

There was no significant association between time spent on diabetes self-care and HSR in models without interaction. Significant interaction term was found between the SF-12 PCS score and time spent on self-care. In models with interaction self-care time has a small, but significant impact on the HSR. In particular, for a PCS score under 40, more time increases the chance to rate the health state as “good”, while for a PCS score above 40 there is a reverse effect.

**Conclusions:**

The additional impact of self-care time on HSR in our sample is small and seems to interact with physical health-related quality of life. More research is needed on whether inclusion of health-related leisure time changes in the denominator of cost-effectiveness analysis is sufficient.

**Supplementary Information:**

The online version contains supplementary material available at 10.1186/s12955-022-02068-9.

## Background

Time spent engaging in healthy behaviors and self-care is a resource that is essential for maintaining or improving health. Time spent on health-related activities has to be considered particularly in chronic diseases as e.g. diabetes mellitus. Diabetes mellitus affects more than 400 million people worldwide [1]. Diabetes management relies largely on activities carried out by patients, such as glucose self-testing, insulin injections, foot care, dietary changes and exercise. Available studies of diabetes self-management show that patient time costs can be substantial – sometimes larger than direct medical costs of an intervention [2, 3]. Professional diabetes educators estimated that people in a stable phase of diabetes care require over 2 hours per day to complete self-care tasks recommended by the American Diabetes Association [2]. In a population-based study in people with diabetes, mean time for diabetes- self management was estimated to be 149 (119-181) minutes per person per week, accounting to 129 (103-157) hours per person per year. The largest proportion of time spent on self-management was due to lifestyle activities such as dietary changes and exercise [3], which will probably compete with other leisure time activities and may affect quality of life as well as willingness to engage in and adhere to self-care behaviour.

Considering increasing expenditures needed for health care systems, economic evaluations have become more important in order to inform decision makers whether current and new technologies are efficient. Often, costs and effects of health care interventions are compared using cost–utility analysis (CUA), that means the evaluation of additional cost per quality-adjusted life-years (QALYs) gained. One of the ongoing methodological debates in the context of economic evaluation is related to the incorporation of different elements of health-related patient time use into the analysis [4–11]. The US Panel on Cost-Effectiveness in Health and Medicine recommended that patient time spent in seeking care and treatment should be included as a cost, i. e. in the numerator of the cost-effectiveness (CE) ratio [4]. The Panel also recommended that health-related changes in time spent on paid or unpaid work and leisure should be included in the denominator of the CE ratio because they are implicitly considered by respondents of health state valuations. Thus for “the reference case analysis, health-related quality of life should be captured by an instrument that, at minimum, implicitly incorporates the effects of morbidity on productivity and leisure”.

Unlike the controversy regarding productivity costs, there is a broad consensus that leisure effects of ill-health *should be* included in the denominator of the CE ratio [4, 10, 11]. However, the amount of empirical research on whether respondents in fact include health-related leisure time changes into health state valuations is limited and the results are inconclusive [12–15]. Therefore, more knowledge on what respondents include in health state valuations and how this affects the subsequent results is required.

In this exploratory study, we have considered the ways in which time spent on diabetes self-care might explain the ratings of the health state in addition to the effects of physical and mental health-related quality of life assessed by the SF-12 questionnaire.

## Methods

### Study design and population

Study design and population have been described in detail elsewhere [16]. Briefly, our cross-sectional study was performed within the KORA FF4 study, the second follow-up of the KORA S4 study (KORA: Cooperative Health Research in the Region of Augsburg). The S4 study is a population-based health survey conducted in the city of Augsburg and two neighbouring counties between 1999 and 2001. A total sample of 6640 participants was drawn from the target population which included all German residents of the region aged 25 to 74 years [17]. Of the 4261 participants in the S4 baseline study (64% response), 3319 persons were eligible for the 14-year follow-up FF4 study conducted from June 2013 to September 2014, and 2279 participated (follow-up response rate of 68.7%). All participants received a comprehensive standardised clinical investigation, interviews and questionnaires [17].

When visiting the KORA study centre, participants were asked if they had been diagnosed with diabetes by a physician or whether they received glucose-lowering medication and, if so, which type of diabetes they had. If there was a diabetes indication (self-report, oral antidiabetic medication) participants’ general practitioners were contacted to validate the diagnosis and the self-reported diabetes type [17]. Of the 2279 participants, 227 (10.0%) were identified as having diagnosed type 2 diabetes. These participants were asked to complete the questionnaire to assess diabetes self-care time as described below, as well as the SF-12 questionnaire which assesses health-related quality of life. One hundred ninety-two participants who had no missing values in the SF-12 questionnaire and less than or equal to 3 missing values in the self-care time questionnaire were included in our analyses (for details regarding missing values see below). We excluded two outliers in patient time (1365 and 1520 minutes per week) so that 190 participants were included in the final analysis.

### Concept of the analysis

We use regression analyses to explain health state ratings in terms of the SF-12 physical and mental component summary (PCS and MCS) scores, time spent on diabetes self-care and a number of background variables. We assume that if time spent on diabetes self-care implicitely plays an important role in health state valuations, we should see this additional effect in regression models. It is important to note that direct (positive) impact of self-care on the physical and mental ability to perform everyday activities and enjoy leisure time is probably captured by the SF-12 questionnaire and we were interested specifically in investigating the influence of the time aspect of diabetes self-care on health state ratings.

### Instruments and variables

The main variables for our analysis were self-reported rating of health state in general, physical and mental health-related quality of life and time spent on diabetes self-care activities. Further variables were included to describe the population and explore possible confounders.

#### General health state

People were asked: ‘How would you rate your health state in general?’ (first question of the SF-12). The question could be answered in five categories: excellent, very good, good, fair, poor.

#### Health-related quality of life

The short form 12 questionnaire (SF-12) is a well established and comprehensively evaluated generic instrument to assess health-related quality of life [18]. It includes 12 items in 8 categories, which can be summed up to a physical component summary score (PCS: general health, physical functioning, physical role, pain) and a mental component summary score (MCS: emotional role, psychosocial wellbeing, vitality, social functioning). The SF-12 version 2 was used in our study. We applied the license from the Hogrefe Publishing [19], which includes the scoring algorithm for the SF-12 based on German data.

#### Time spent on diabetes self-care

Time spent on self-care activities was measured using a questionnaire developed on the basis of instruments assessing self-care behaviour in people with diabetes. The questionnaire was also evaluated in focus groups and was tested in a random sample of people with type 2 diabetes [20]. The questionnaire asked participants if they had spent time on particular diabetes-related self-management activities during the last 7 days. The questionnaire included disease-specific clinical activities such as measuring blood glucose and blood pressure, taking medication (insulin or oral antihyperglycaemic drugs or other antihyperglycaemic drugs by injection), foot and skin care, shopping for medication and specific health items, decision-making, information seeking, and lifestyle-related activities such as exercise, shopping and cooking or other diabetes-related activities. Respondents were asked 1) whether they spent time on particular diabetes-related self-care activities and 2) how many hours and/or minutes they spent on them. To cover non-daily self-care activities, the question referred to the previous 7 days. All time variables are in minutes per week. Finally, overall patient time spent on diabetes self-care activities was calculated as the sum of all 14 time variables.

#### Further variables

To describe the population and adjust for possible confounders, data on demographic, socioeconomic, clinical and lifestyle factors of the 190 participants were assessed. *Demographic and socioeconomic variables* included: age, sex, employment status (yes/no), education (low, middle, high) and living together with a partner (yes/no). *Diabetes specific measures* included: HbA1c (investigation in the KORA study center), diabetes duration and type of treatment (insulin and/or oral antihyperglycaemic medication) (self-reported and validated by the participants’ physicians), diabetes-related complications (at least one of the following: retinopathy, retinal detachment, blindness, proteinuria, dialysis, infectious kidney or bladder disease, kidney transplant, end-stage renal disease, peripheral vascular disease, and lower extremity amputation; all self-reported), and diabetes-related distress (PAID) [21]. *Measures of general health* included comorbidities (myocardial infarction, angina pectoris, stroke, cancer, all self-reported). *Lifestyle indicators* included: smoking status (current, former or never, self-reported) and body mass index (BMI, clinical investigation).

### Statistical analysis

#### Descriptive analyses

Descriptive statistics are provided as frequencies and percentages or mean values, standard deviations, medians and interquartile ranges (IQR) depending on the nature of considered variables.

#### Missing values in time variables

Six persons had one missing value in answers to the 14 questions whether they spent time on particular diabetes-related activities, and one person three missing values in these answers. In these cases, missing values were set to ‘0’ (no time) in our analysis. Twenty three persons had in total 44 missing values in time variables related to particular self-care activities although they answered the corresponding question with “yes”. In our main analysis we set all individual missing time variables to zero, while in the sensitivity analysis we imputed the corresponding medians based on non-missing values. We believe that missing values in time variables occurred rather for smaller values (i.e. missings not at random) so that our two approaches may represent two extreme scenarios.

#### Regression analyses

Regression models were fitted to evaluate the impact of time spent on self-care on the rating of the general health status beyond the physical and the mental score of the SF-12. We modelled the dichotomized version (“Excellent/Very good/Good: good”, “Fair/Poor: poor”) of the general health state ratings using multiple logistic regression with the following independent variables: PCS and MCS of the SF12 and time spent on self-care, cf. model (i) in Table 2. To study the impact of potential confounders we considered futher multiple logistic regression models with additional independent variables: age and sex (cf., model (ii) in Table 2) and age, sex, school education, employment status,living together with a partner, cf. model (iii) in Table 2. The corresponding sensitivity analysis (with imputed medians for missing time variables related to particular self-care activities) is shown in Table SA2. To get a deeper insight into the dependency structure between the variables in model (i) we studied whether there exists an interaction between PCS and MCS scores of the SF-12 and time spent on diabetes self-care. By means of backword selection we found out which terms had a significant impact on the rating of the general health state. Thereby, we considered different treatments of missing values (main and sensitivity analyses as described above), cf. models (ia) and (ib) in Table 3.

The significance level was set to α = 0.05.

SAS software, V.9.4 (SAS Institute Inc., Cary, NC, USA) was used for statistical analysis.

## Results

The sample (*n* = 190) is described in detail in Table 1. The mean age was 69 years, 60% of the sample were men. Two thirds of participants had low school education. 23% of participants were employed.

**Table 1:** Description of the study population

Characteristics	Mean ± SD, Median (Q1-Q3) or Frequency (%)
**Demographic and sociodemographic characteristics**	
Age (years)	69.2 ± 10.1, 71.0 (63-77)
Gender (male)	114 (60.0%)
Employment status (yes)	44 (23.2%)
School education	
High (Abitur/Fachabitur/Fachhochschulreife)	31 (16.3%)
Middle (Mittlere Reife/Realschule)	34 (17.9%)
Low (Hauptschulabschluss)	125 (65.8%)
Living together with a partner (yes)	138 (72.6%)
**Diabetes specific characteristics**	
Diabetes duration (*n* = 175) (years)	10.5 ± 8.2, 8.0 (5.0-14.0)
Type of treatment (*n* = 189)	
No antihyperglycaemic medication	30 (15.9%)
Insulin only	10 (5.3%)
Oral antihyperglycaemic medication only	130 (68.8%)
Insulin and oral antihyperglycaemic medication	19 (10.1%)
HbA1c (%) (*n* = 188)	6.7 ± 1.1, 6.5 (6.0-7.1)
< 6.5%	90 (47.9%)
6.5 to < 7.5%	65 (34.6%)
≥ 7.5%	33 (17.6%)
Diabetes-related complications (at least one of 11)	90 (47.4%)
PAID (*n* = 182)	32.3 ± 13.1, 27.5 (21-40)
**Lifestyle**	
Current Smoker	18 (9.5%)
BMI (kg/m^2^)	30.9 ± 5.2, 30.4 (27.2-34.3)
< 25 kg/m^2^	21 (11.1%)
25- < 30 kg/m^2^	67 (35.3%)
30- < 35 kg/m^2^	61 (32.1%)
≥ 35 kg/m^2^	41 (21.6%)
**Comorbidities**	
Myocardial infarction	23 (12.1%)
Angina pectoris	16 (8.4%)
Stroke (n = 189)	11 (5.8%)
Cancer	30 (15.8%)
**General health status, QoL and patient time**	
Rating of the general health status	
Excellent	1 (0.5%)
Very good	12 (6.3%)
Good	135 (71.1%)
Fair	38 (20%)
Poor	4 (2.1%)
QoL: Physical score of SF12	43.2 ± 9.8, 44.5 (35.8-51.3)
QoL: Mental score of SF12	51.6 ± 9.6, 54.3 (46.5-57.9)
Patient time (hours per week)	2.0 ± 3.1, 0.7 (0.2-2.6)
Patient time (hours per week) with imputations	2.1 ± 3.2, 0.8 (0.2-2.8)

Q1 the 25%-quartile, Q3 the 75%-Quartile

n = 190 patients unless indicated otherwise

*BMI* body mass index

Median SF12 PCS score was 44.5 and median MCS score was 54.3. The majority of the participants rated their general health state as “good” (71%), 21% rated it as “fair. Mean time spent on self-management activities was about 2 hours per week.

Table 2 displays the results of the multiple logistic regression models. The estimated Odds Ratio (OR) to rate the general health status as “good” was 1.23 (95%-CI 1.15-1.32) for a one point increase in the SF12 PCS score and 1.14 (95%-CI 1.08-1.20) for a one point increase in the MCS score, cf. model (i). Both associations were highly significant (*p*-value < 0.0001). However, time spent on self-care was not significantly associated with the general health status (OR = 1.17, *p*-value 0.0692) in this model. Models (ii) and (iii) indicate that none of the considered factors like age, sex, employment status, partner or school education changed the association between time spent on self-care and general health state. The same results were obtained in the sensitivity analysis with imputed medians for missing values in time variables, cf. Table A2.

**Table 2:** Odds ratios to rate the general health state as “good”

	Model (i)		Model (ii)		Model (iii)	
	OR (***p***-value)	95%-CI	OR (***p***-value)	95%-CI	OR (***p***-value)	95%-CI
QoL: Physical score of SF12 per 1 unit increase	**1.23** (< 0.0001)	(1.15,1.32)	**1.24** (< 0.0001)	(1.15,1.33)	**1.24** (< 0.0001)	(1.15,1.34)
QoL: Mental score of SF12 per 1 unit increase	**1.14** (< 0.0001)	(1.08,1.20)	**1.13** (< 0.0001)	(1.07,1.20)	**1.13** (< 0.0001)	(1.06,1.19)
Patient time (per 1 hour/week increase)	1.17 (0.0692)	(0.99,1.38)	1.16 (0.0898)	(0.98,1.38)	1.19 (0.0600)	(0.99,1.44)
Age (per 1 year increase)			1.03 (0.3738)	(0.97,1.08)	1.04 (0.1967)	(0.98,1.12)
Sex (female vs. male)			0.66 (0.4375)	(0.23,1.88)	0.67 (0.4591)	(0.23,1.95)
Employment status (yes vs. no)					2.83 (0.2660)	(0.45,17.74)
Partner (yes vs. no)					0.68 (0.5575)	(0.19,2.46)
School education (high/middle vs. low)					1.67 (0.4094)	(0.49,5.64)

Max-rescaled R-square was 0.64 in (i) and (ii) and 0.65 in (iii)

Area Under the Curve (AUC) was 0.93 in (i), 0.94 in (ii) and (iii)

Model (i) without confounder. Model (ii) with adjustment for age and sex. Model (iii) with further adjustment for school education, employment status,living together with a partner

*CI* Confidence Intervalls

Models with interaction terms between the SF-12 PCS and MCS scores and time spent on self-care showed a significant interaction between physical scores of SF-12 and time spent on self-care. Table 3 shows the regression coefficients (β’s) of multiple logistic models: (i) without interaction, (ia) with interaction and (ib) with interaction and imputed median values for missings in time variables. In both models coefficients related to patient time as well as the interaction were significantly associated with the general health state as “good”. Based on regression coefficients of patient time and interaction term one can easily calculate that for SF12 PCS values under 40, higher patient time increases the chance to rate the health status as “good”, while for PCS values above 40 patient time had a reverse effect.

**Table 3:** Regression coefficients and odds ratios to rate the general health state as “good”^b^

	Model (i)		Model (ia)		Model (ib)^a^	
	β (***p***-value) 95%-CI	OR 95%-CI	β (***p***-value) 95%-CI	OR 95%-CI	β (***p***-value) 95%-CI	OR 95%-CI
QoL: Physical score of SF12	**0.2083** (< 0.0001) (0.1374,0.2793)	1.23 (1.15,1.32)	**0.2693** (< 0.0001) (0.1748,0.3637)		**0.2684** (< 0.0001) (0.1747,0.3622)	
QoL: Mental score of SF12	**0.1270** (< 0.0001) (0.0725,0.1816)	1.14 (1.08,1.20)	**0.1350** (< 0.0001) (0.0770,0.1930)	1.14 (1.08,1.21)	**0.1341** (< 0.0001) (0.0764,0.1918)	1.14 (1.08,1.21)
Patient time (hour per week)	0.1566 (0.0692) (−0.0123,0.3255)	1.17 (0.99,1.38)	**0.8844** (0.0018) (0.3288,1.4400)		**0.8375** (0.0025) (0.2955,1.3795)	
Interaction between Physical score of SF12 and patient time			**−0.0220** (0.0029) (− 0.0365,-0.0075)		**− 0.0211** (0.0040) (− 0.0354,-0.0067)	

OR = exp.(β)

Max-rescaled R-square was 0.64 in (i), 0.67 in (ia) and (ib)

Area Under the Curve was 0.93 in (i), 0.94 in (ia) and (ib)

^a^Sensitivity analysis: missing values in time variables related to particular self-care activities were replaced by the corresponding medians

^b^models with and without interaction term

**Fig. 1:**
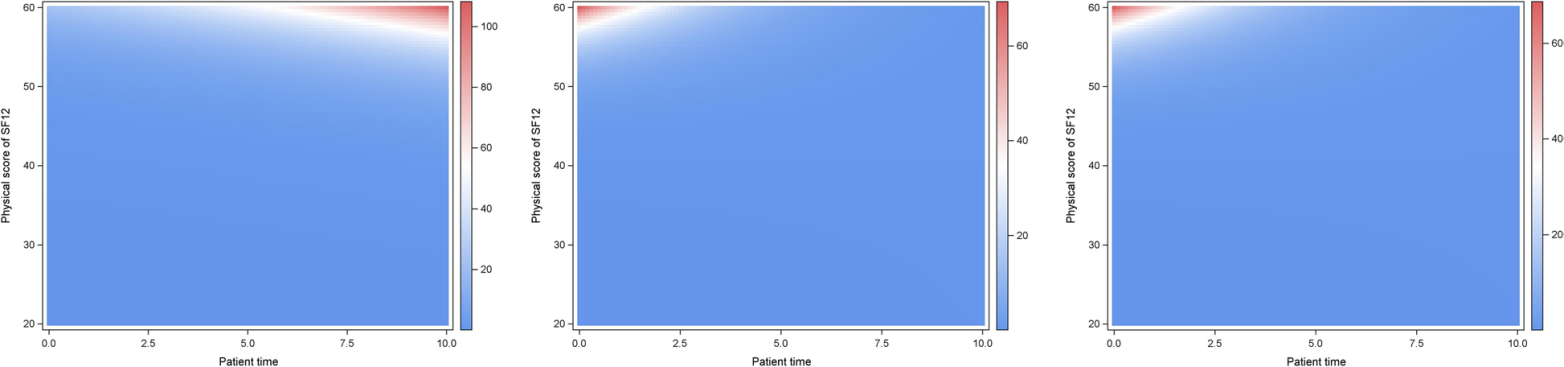
ORs to rate of the general health state as “good” (models (i), (ia) and (ib), from left to right). Shows ORs to rate general health state as “good” for a person with given SF 12 PCS score (y-axis) and patient time (x-axis) compared to a person with median PCS score (44.5) and median patient time (0.7 hour per week) for models (i), (ia) and (ib)

**Fig. 2:**
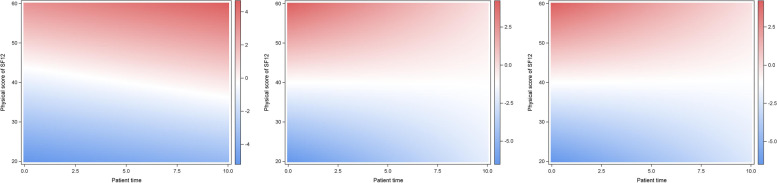
Effects on the log-scale (i.e., log (OR)) to rate the general health state as”good” (models (i), (ia) and (ib), from left to right). OR-values (or log (OR)-values) for a person with patient time given on x-axis and physical score of SF12 given on y-axis compared to a person with median patient time (44.5) and median physical score of SF12 (44.5) are presented by different colors corresponding to color scale on the right of each graph

Figure 1 shows ORs to rate general health state as “good” for a person with given SF 12 PCS score (y-axis) and patient time (x-axis) compared to a person with median PCS score (44.5) and median patient time (0.7 hour per week) for models (i), (ia) and (ib). Estimated ORs are smaller in models with interaction (ia) and (ib) than in model (i) without interaction. Both models with interaction, i.e., (ia) and (ib), yield very similar results despite different handling of missing values. Figure 2 shows the same ORs on the log-scale.

## Discussion

### Main findings

In this study we explored a possible association between time spent on diabetes self-care and the rating of the health state using regression models. We assumed that if time spent on diabetes self-care leads to lost leisure or other relevant experiences, respondents would (implicitly) consider it in their health state ratings in addition to other physical or mental aspects as measured by the SF-12 questionnaire.

We did not observe any additional effect of self-care time on the health state ratings in regression models without interaction. However, we observed a significant interaction between SF-12 PCS score and time spent on self-care regarding health state ratings. In particular, for PCS values over 40, more self-care time decreases the chance to rate a health state as “good”. That means that models with interaction imply that persons with highest PCS score of SF-12 and rather low self-care time have the highest chance to rate their health state as “excellent, very good or good”.

Even though the additional effect of self-care time on the health state rating is small, the finding is in line with the assumption that time spent on diabetes self-care competes with other leisure activities and may negatively influence self-rated health. The observed positive effect of self-care time on the rating of health state in respondents with physical score of SF-12 under 40 may be explained by less “competition” between self-care and other leisure activities in the state of poor health.

### Comparison to other studies

The amount of empirical research on whether respondents include leisure effects of ill-health into health state valuations is limited. Available studies [13–15] were conducted among respondents from the general public who were asked to value health states on a visual analogue scale (VAS) or using a time trade-off (TTO) method. The respondents were asked afterwards whether they had considered leisure time effects of ill health in their valuations.

The majority of respondents (61-88%) in available studies stated to spontaneously consider health-related changes in leisure in their health state valuations. However, it is unclear whether this leads to an adequate valuation of lost leisure across various health states: In a study by Brouwer et al., the incorporation of leisure proved to be influential in the valuation with the visual analogue scale (VAS), but only for the most severe health state [14]. In a study by Krol et al. using a similar design, there were no significant differences between the valuations of respondents who included or excluded effects on leisure time [13]. In another study by Krol et al. using TTO instead of VAS, respondents including leisure time gave lower TTO values to the three health states than respondents who had not included leisure time. The differences, however, were only significant for one health state out of three [15].

Our respondents rated their own health states and we chose to model the possible effect of lost leisure using data on diabetes self-care time which was available for them. Our results are in line with previous studies and suggest that respondents implicitly incorporate time aspects of diabetes self-care in their health state ratings, but this effect is rather small and may even be heterogenous.

### Strengths and limitations

Before discussing the implications of our findings, we need to stress that our study was based on a relatively small, but population-based sample of people with diabetes. Diabetes was identified by self-report or antihyperglycemic medication, however, participants’ general practitioners were contacted to validate the diagnosis and the self-reported diabetes type [17]. Health state was rated using a 5-point Likert-type scale, which may be less sensitive to changes in leisure time compared to VAS or TTO valuations Time spent on diabetes self-care was assessed using retrospective questions, which may lead to overestimation or underestimation of the actual time use because of recall bias.

An important limitation of our study is that we cannot exclude the possibility that respondents implicitly considered time aspects of diabetes self-care when answering the SF-12 questionnaire. However, this is unlikely because the SF-12 focuses on function and abilities rather than on other aspects related to ill health such as the lost leisure time. Time spent on diabetes self-care is only one aspect of leisure time effects due to ill-health. Moreover, lost leisure time because of diabetes self-care may be less of a problem for our respondents (mean age 69 years old) compared to the younger people with more competing demands for time.

## Conclusions and implications for further research

The impact of self-care time in our sample – if respondents indeed implicitly incorporate it into their ratings of the health state – is small and more complex than a simple linear association. If leisure time effects of chronic illness should be incorporated into economic evaluation, further research is warranted to ensure that current practice gives sufficient weight to changes in leisure time due to changes in health. Studies in larger samples including participants of different age and also using other health state valuation techniques, such as VAS and TTO may be useful. The important general point here is that the focus of attention that drives our preferences in health states valuations is different from the focus of attention that explains the intensity of our experiences [22]. Specifically, one has to ask whether inclusion of leisure time effects by means of preference-based health-related quality of life instruments in the denominator of the cost-effectiveness ratio is sufficient or whether new ways (e.g. measures of experienced utility or monetary valuation) to include leisure in economic evaluations have to be found.

## Supplementary Information

**Additional file 1: Table SA2.** Odds ratios to rate the general health state as “good” – sensitivity analysis.

## Data Availability

Data may be obtained from a third party and are not publicly available. Project agreements to use and access KORA data can be requested from national and international researchers via the KORA-PASST tool under https://epi.helmholtz-muenchen.de/.

## Abbreviations

BMI: Body mass index
CE: Cost-effectiveness
CUA: Cost-utility analysis
HSR: Health state rating
IQR: Interquartile ranges
MCS: Mental component summary
OR: Odds Ratio
PAID: Diabetes-related distress
PCS: Physical component summary
QALYs: Quality-adjusted life-years
TTO: Time trade-off
VAS: Visual analogue scale
