# Supplementary material for: Self-care time and rating of health state in persons with diabetes: results from the population-based KORA survey in Germany
Source: Health Qual Life Outcomes. 2022 Dec 14;20:163. doi: 10.1186/s12955-022-02068-9 (PMC9749283; doi:10.1186/s12955-022-02068-9)
Supplement: Supplementary file 1 — Additional file 1: Table SA2. Odds ratios to rate the general health state as “good” – sensitivity analysis. [file 12955_2022_2068_MOESM1_ESM.docx]

**Table SA2: Odds ratios to rate the general health state as “good” – sensitivity analysis**

|  | **Model (i)** | | **Model (ii)** | | **Model (iii)** | |
| --- | --- | --- | --- | --- | --- | --- |
|  | **OR  (p-value)** | **95%-CI** | **OR  (p-value)** | **95%-CI** | **OR  (p-value)** | **95%-CI** |
| QoL: Physical score of SF12 | **1.23** (<0.0001) | (1.15,1.32) | **1.24** (<0.0001) | (1.15,1.33) | **1.25** (<0.0001) | (1.15,1.34) |
| QoL: Mental score of SF12 | **1.14** (<0.0001) | (1.08,1.20) | **1.13** (<0.0001) | (1.07,1.20) | **1.13** (<0.0001) | (1.06,1.19) |
| Patient time (hour per week) | 1.17 (0.0534) | (1.00,1.36) | 1.16 (0.0732) | (0.99,1.36) | **1.19** (0.0484) | (1.00,1.41) |
| Age (years) |  |  | 1.02 (0.3950) | (0.97,1.08) | 1.04 (0.2130) | (0.98,1.11) |
| Sex (female vs. male) |  |  | 0.66 (0.4427) | (0.23,1.89) | 0.67 (0.4688) | (0.23,1.97) |
| Employment status (yes vs. no) |  |  |  |  | 2.82 (0.2680) | (0.45,17.71) |
| Partner (yes vs. no) |  |  |  |  | 0.67 (0.5437) | (0.19,2.42) |
| School education  (high/middle vs. low) |  |  |  |  | 1.72 (0.3839) | (0.51,5.84) |

CI Confidence Intervals
Max-rescaled R-square was 0.64 in (i) and (ii) and 0.66 in (iii)

Area Under the Curve (AUC) was 0.93 in (i), 0.94 in (ii) and (iii)

Sensitivity analysis with imputed medians for missing values in time variables
